# PP54 Next Generation Sequencing Gene Panel For Medical Lung Cancer Medical Care

**DOI:** 10.1017/S0266462325102730

**Published:** 2025-12-29

**Authors:** Melissa Bougas, Marie Daclin, Denis-Jean David, Cédric Carbonneil

## Abstract

**Introduction:**

The objective of this assessment was to determine the benefit of using a next-generation sequencing gene panel for the clinical management of patients with non-small cell lung cancer. The aim was to assess the diagnostic performance, identify the molecular alterations of interest, and define the role of this technology in the therapeutic care of these patients.

**Methods:**

The method used for this assessment were based on: (i) a critical analysis of systematic reviews and meta-analyses and clinical practice guidelines identified by a systematic search based on PICO criteria (Population, Intervention, Comparator, Outcome); (ii) the identification of the level of evidence of the clinical actionability of molecular targets, as set out by the European Society for Medical Oncology scale, and targeted therapies included on the list of reimbursable drugs, or drugs that have compassionate use authorizations; and (iii) stakeholder consultations, as well as public health institutions.

**Results:**

Assessment of the evidence and data demonstrated that next-generation sequencing gene panel testing (EGFR, ALK, ROS1, BRAF, RET, and KRAS): (i) is highly concordant with the comparators (Kappa coefficient of 0.884); (ii) can detect additional targetable molecular alterations (marginal increase in the detection capacity of 2%); (iii) makes better use of the tumor tissue; and (iv) has clinical utility, demonstrated by the benefits provided by targeted therapies.

**Conclusions:**

The next-generation sequencing gene panel (EGFR, ALK, ROS1, BRAF, RET, KRAS) was recommended for patients with locally advanced or metastatic non-small cell lung cancer, following diagnosis or in cases of progression, excluding any emergency situations. The composition of the gene panel may be subject to change, in accordance with favorable assessments of new gene alterations.

